# Prophylactic effect of negative‐pressure wound therapy and delayed sutures against incisional‐surgical site infection after emergency laparotomy for colorectal perforation: A multicenter retrospective cohort study

**DOI:** 10.1002/ags3.12643

**Published:** 2022-11-27

**Authors:** Keita Nakatsutsumi, Akira Endo, Hiroshi Asano, Shoichi Shinohara, Ryo Kurosaki, Shuji Kawashima, Wataru Ishii, Masashi Nozawa, Nobumi Tagaya, Yasuhiro Otomo

**Affiliations:** ^1^ Trauma and Acute Critical Care Center Tokyo Medical and Dental University Hospital Tokyo Japan; ^2^ Department of Acute Critical Care Medicine Tsuchiura Kyodo General Hospital Ibaraki Japan; ^3^ Department of General Surgery Saitama Medical University Saitama Japan; ^4^ Department of Surgery, Division of Gastroenterological, General and Transplant Surgery Jichi Medical University Tochigi Japan; ^5^ Surgery Department Japanese Red Cross Maebashi Hospital Maebashi Japan; ^6^ Department of Emergency and Critical Care Medicine Wakayama Medical University Wakayama Japan; ^7^ Department of Emergency Medicine Japanese Red Cross Society Kyoto Daini Hospital Kyoto Japan; ^8^ Department of Surgery Shimada General Medical Center Shizuoka Japan; ^9^ Department of Surgery Itabashi Chuo Medical Center Tokyo Japan; ^10^ Department of Acute Critical Care and Disaster Medicine, Graduate School of Medical and Dental Sciences Tokyo Medical and Dental University Tokyo Japan

**Keywords:** colorectal perforation, emergency surgery, incisional‐surgical site infection, negative‐pressure wound therapy, prophylactic treatment

## Abstract

**Aim:**

The prophylactic effect of negative‐pressure wound therapy against incisional surgical site infection after highly contaminated laparotomies has not been sufficiently explored. This study aimed to evaluate the prophylactic effect of negative‐pressure wound therapy against incisional surgical site infection after emergency surgery for colorectal perforation.

**Methods:**

This nationwide, multicenter, retrospective cohort study analyzed data from the 48 emergency hospitals certificated by the Japanese Society for Abdominal Emergency Medicine. Patients who underwent an emergency laparotomy for colorectal perforation between April 2015 and March 2020 were included in this study. Outcomes, including the incidence of incisional surgical site infection, were compared between patients who were treated with prophylactic negative‐pressure wound therapy and delayed sutures (i.e., negative‐pressure wound therapy group) and patients who were treated with regular wound management (i.e., control group) using 1:4 propensity score matching analysis.

**Results:**

The negative‐pressure wound therapy group comprised 88 patients, whereas the control group consisted of 1535 patients. Of them, 82 propensity score‐matched pairs (negative‐pressure wound therapy group: 82; control group: 328) were evaluated. The negative‐pressure wound therapy group showed a lower incidence of incisional surgical site infection [18 (22.0%) in the negative‐pressure wound therapy group and 115 (35.0%) in the control group, odds ratio, 0.52; 95% confidence interval, 0.30 to 0.92; *p* = 0.026].

**Conclusions:**

The prophylactic use of negative‐pressure wound therapy with delayed sutures was associated with a lower incidence of incisional surgical site infection after emergency surgery for colorectal perforation.

## Graphical abstract

The prophylactic use of negative‐pressure wound therapy was associated with a lower incidence of incisional surgical site infection after emergency surgery for colorectal perforation. Patients without diabetes mellitus or with longer operative times (≥190 min) might benefit from negative pressure wound therapy.

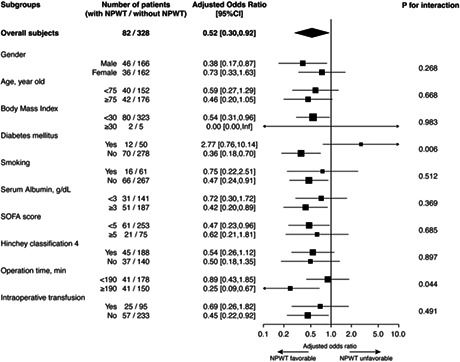

## 1 INTRODUCTION

Colorectal perforation is common and associated with high morbidity and mortality.^1^ Emergency surgery for colorectal perforation is associated with a high risk of incisional surgical site infection (iSSI).^2^, ^3^ In addition, some studies have advocated that iSSI could increase the length of hospitalization, healthcare costs, and mortality.^4^, ^5^ Therefore, preventing iSSI is important to improve the outcomes of patients with colorectal perforation undergoing emergency surgery.

Negative‐pressure wound therapy (NPWT) was initially developed as a treatment for chronic and difficult‐to‐manage wounds, such as infected bedsores.^6^ Over recent years, as the prophylactic effect of NPWT against iSSI has drawn much attention, NPWT has been widely used prophylactically in several types of clean surgeries, including vascular, gynecologic, and orthopaedic surgeries.^7^, ^8^, ^9^ For laparotomies, the prophylactic use of NPWT is also gradually expanding; however, its evidence is not sufficiently evaluated. Therefore, the WHO guidelines for the prevention of SSI only provide limited recommendation for the prophylactic use of NPWT.^10^, ^11^ Moreover, no large‐scale study has investigated the prophylactic use of NPWT for highly contaminated laparotomies, such as surgery for colorectal perforation, and adequate indications for the prophylactic use of NPWT remain unclear.

The present study aimed to evaluate the prophylactic effect of NPWT against iSSI after emergency surgery for colorectal perforation, by sufficient analysis using multicenter data. These results could contribute to improving the outcomes of critically ill patients with colorectal perforation.

## 2 METHODS

### 2.1 Study design and settings

This nationwide, multicenter, retrospective cohort study evaluated the prophylactic effect of NPWT against iSSI after emergency surgery for colorectal perforation. Data were collected from 48 emergency hospitals certificated by the Japanese Society for Abdominal Emergency Medicine. The patients were divided into two groups—namely, (1) the NPWT group, which comprised patients who were treated with prophylactic NPWT and delayed sutures, and (2) the control group, which consisted of patients who were treated with regular wound management. Propensity score matching analysis was performed to explain the differences in clinical backgrounds and compare the outcomes between the two groups. Subpopulations that would benefit from the prophylactic use of NPWT were also explored by assessing the interaction effects between NPWT and patient characteristics.

This study was conducted in accordance with the principles embodied in the Declaration of Helsinki (as revised in Fortaleza, Brazil, in October 2013) and complied with STROBE‐guidelines (https://strobe‐statement.org/). The ethics committee of the Tokyo Medical and Dental University approved this study (approval no.: M2020‐125).

### 2.2 Study population

Patients who underwent emergency surgery for colorectal perforation between April 2015 and March 2020 were included in this study. Patients who met at least one of the following criteria were excluded: (1) laparoscopic surgery, (2) open abdominal management, or (3) multiple laparotomies during the hospitalization.

### 2.3 Data collection and outcomes

The following patient data were collected: basic information (age, sex, body mass index [BMI], performance status, past medical history), preoperative vital signs (blood pressure, heart rate, respiratory rate, Glasgow coma scale), preoperative blood examination results (white blood cell count, C‐reactive protein [CRP], albumin, and lactate levels, creatinine clearance, base excess, and partial pressure of oxygen in arterial blood [PaO_2_]), preoperative Sequential Organ Failure Assessment (SOFA) score, surgery‐related information (Hinchey classification, operative time, incidence of intraoperative transfusion, and operative procedure), postoperative complications, status at hospital discharge, length of hospital stay, and healthcare costs. Hinchey classification, generally used for acute diverticulitis, was applied to evaluate the extent of peritonitis in the present study because this classification was useful and convenient as the common definition of degree of intra‐abdominal contamination among surgeons.^12^

The primary outcome was the incidence of iSSI. The secondary outcomes were the length of hospital stay, healthcare costs (in USD), in‐hospital deaths, and incidence of complications, except for iSSI.

### 2.4 Definitions and procedures

iSSI was diagnosed by the attending surgeons according to the surgical site infection criteria of the Centers for Disease Control and Prevention (CDC).^13^ The indication of NPWT had not yet been established. Therefore, each attending surgeon determined the requirement for NPWT based on the degree of contamination, risk factors of patients, and accessibility of NPWT. Patients in the NPWT group received NPWT for their open wound immediately after surgery, using a commercially available NPWT system (V.A.C.® Therapy, KCI, San Antonio, TX or RENASYS™, Smith and Nephew, Minato‐ku, Tokyo) and the dressing was changed every 48–72 h. When the attending surgeons found the wound to be suitable for closing (no wound contamination and good development of granulation tissue), NPWT was discontinued, and delayed suturing was performed at the bedside. After closing the wound, regular wound management was continued at each facility. In contrast, patients in the control group underwent primary closure at the end of surgery, and regular wound management was continued. The total costs during hospitalization were assessed using Japanese yen, and they were presented as US dollars at an exchange rate of 120 yen to a dollar.

### 2.5 Statistical analysis

The missing data were supplied using the multiple imputation method by the chained equation, to maximize the use of available data. In all 15 datasets, 10 iterations were performed using the package “mice.”^14^ Descriptive statistics were reported as counts and percentages for categorical variables and as medians and the 25th–75th percentiles for numeric or ordered variables after all imputed datasets were gathered into one dataset. Predictive statistics were reported as point estimations and 95% confidence intervals (CI) integrated across the imputed datasets, according to Rubin's rule.^15^

Propensity score matching analysis was performed to consider the unbalanced characteristics between the two groups.^16^ The propensity score predicting NPWT for each patient was estimated by logistic regression analysis based on patient's basic information (age, BMI, performance status score [≥3 or not], current smoking status, comorbidities [diabetes mellitus, cancer, diseases requiring immunosuppressive therapy]), preoperative status (mean blood pressure, respiratory rate, SOFA score), preoperative blood examination results (CRP, albumin, and lactate levels), and surgery‐related information (Hinchey grade, operative time, whether or not intraoperative transfusion was performed, whether or not stoma was constructed, and whether or not wound retractor was used). The variables were a priori chosen from a clinical perspective and from subject matter knowledge. The logit‐transformed propensity score was calculated for each imputed dataset and averaged across datasets. Based on these values, 1:4 matched pairs of patients were extracted from the NPWT and control groups. This ratio was set to achieve the maximum use of available data and feasibility of the match balance. The balance of matching between the two groups was evaluated using the absolute standardized mean difference of all the variables, in which values lower than 0.1 were considered acceptable.^17^ The caliper width for matching was set at 0.2 for a well‐matched balance between the two groups. For intergroup comparison of the outcomes with the propensity score‐matched patients, the chi‐square test was performed for categorical variables, whereas Student's *t*‐test was performed for continuous variables.

A multivariate logistic regression model for all patients (not propensity score‐matched patients) was also used to evaluate the prophylactic effect of NPWT by a sensitivity analysis. The covariates in this analysis were the variables used in the propensity score calculation. Multicollinearity was evaluated using the variance inflation factor, with the tolerance value set at <2.

To explore potential subpopulations who were likely to benefit from the prophylactic use of NPWT, the primary outcome was compared in the propensity score‐matched cohort across the subgroups according to sex, age (<75 or ≥75 years), BMI (<30 or ≥30), presence or absence of diabetes mellitus, presence or absence of current smoking, serum albumin level (<3 or ≥3 g/dl), Hinchey grade (<4 or ≥4), operative time (<190 or ≥190 min), and whether intraoperative transfusion was performed. Logistic regression analysis was performed to evaluate the odds ratio (OR) for the interaction term between NPWT and the dichotomized subgroups for the incidence of iSSI.

Statistical analysis was performed using R 4.1.1 (The R Foundation for Statistical Computing, Vienna, Austria), with the level of significance set at *p* < 0.05.

## 3 RESULTS

### 3.1 Study population

**FIGURE 1:**
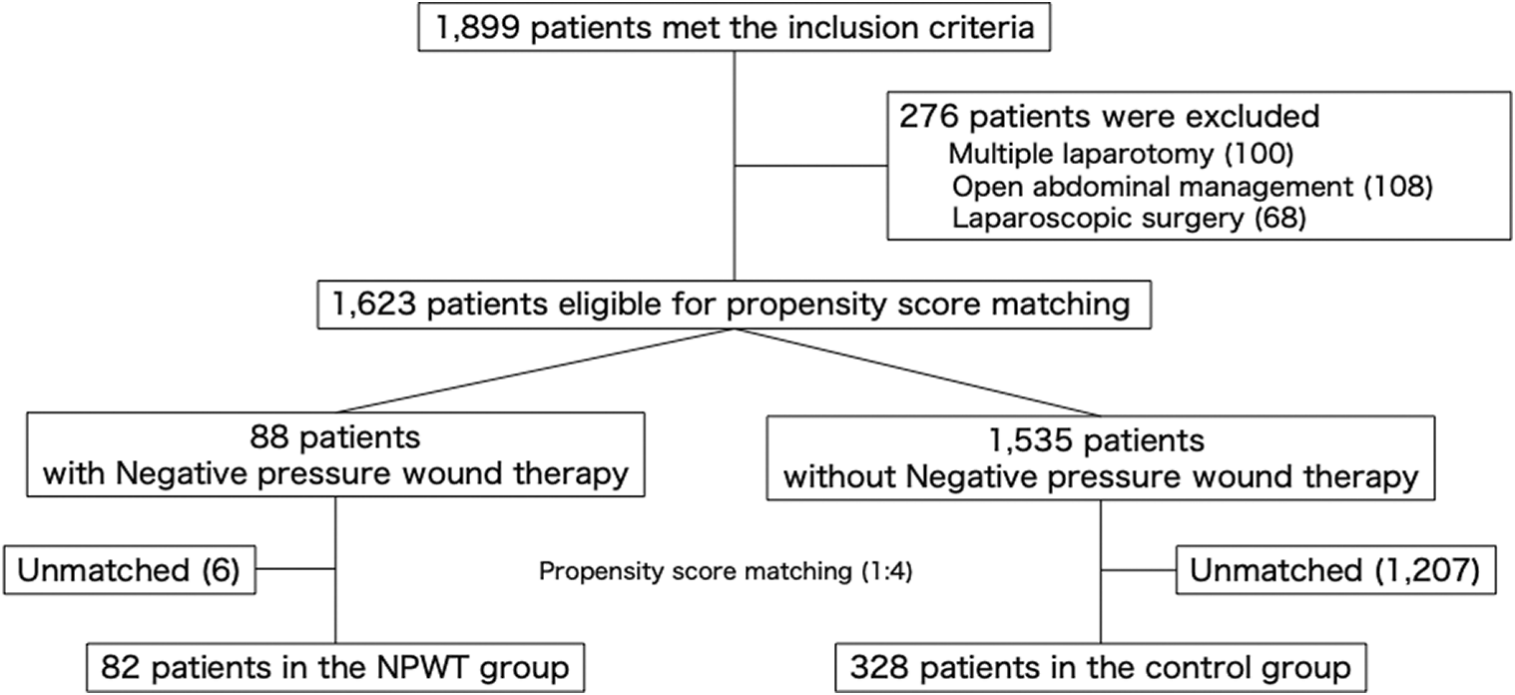
Flow diagram of the patient selection process. NPWT: Negative‐pressure wound therapy

A flow diagram of the patient selection process is shown in Figure 1. A total of 1623 patients were eligible for analysis. The number of cases in each facility are summarized in Table S1. The patients were divided into the NPWT group (*n* = 88) and the control group (*n* = 1535), from which 82 propensity score‐matched pairs (82 and 328 patients for the NPWT and the control groups, respectively) were generated.

### 3.2 Patient characteristics

The all‐patient non‐imputed demographic data are summarized in Table S2. The patients' demographic data in the multiply imputed datasets before and after propensity score matching are summarized in Table 1. Before matching, the serum lactate levels and proportion of patients with Hinchey grade 4 were higher in the NPWT group than in the control group. The standardized mean differences in the variables used in the propensity score estimation indicated a well‐matched balance.

**TABLE 1:** Characteristics of the patients before and after propensity score matching

Variables	Overall study cohort			Propensity score‐matching		
	NPWT (*n* = 88)	Control (*n* = 1535)	SMD	NPWT (*n* = 82)	Control (*n* = 328)	SMD
Age, years	75 (67–80)	75 (65–83)	−0.01	75 (67–81)	76 (67–83)	0.07
Male^a^	50 (56.8)	741 (48.3)	−0.17	46 (56.1)	166 (50.6)	−0.10
Body Mass Index	21 (18–24)	22 (19–24)	0.07	21 (19–25)	21 (19–24)	−0.06
Performance status ≥3	4 (4.5)	252 (16.4)	0.39	4 (4.9)	17 (5.3)	0.02
Smoking	17 (18.9)	306 (20.0)	0.02	16 (19.0)	61 (18.6)	−0.00
Diabetes mellitus	14 (15.9)	201 (13.1)	−0.07	12 (14.6)	50 (15.2)	0.01
Immunosuppressive therapy	7 (8.0)	154 (10)	0.07	7 (8.5)	27 (8.5)	−0.01
Cancer‐bearing	21 (23.9)	383 (25.0)	0.02	20 (24.4)	76 (23.2)	−0.02
Perioperative vital signs						
Median blood pressure, mmHg	92 (79–107)	89 (77–102)	−0.17	92 (78–106)	92 (80–105)	−0.00
Heart rate^a^, beats/min	99 (84–115)	94 (80–110)	−0.17	99 (81–115)	96 (82–110)	−0.00
Respiratory rate, beats/min	20 (18–25)	20 (18–26)	0.12	20 (18–25)	21 (18–26)	0.02
Glasgow coma scale^a^	15 (15–15)	15 (15–15)	−0.04	15 (15–15)	15 (15–15)	−0.06
Perioperative blood exam results						
White blood cell count^a^, 10^3^/μl	7.7 (3.8–12.6)	8.1 (4.2–1.32)	0.02	7.4 (3.8–12.4)	7.3 (3.9–12.7)	−0.04
C‐reactive protein, mg/dl	8.7 (0.3–24.7)	9.6 (0.8–21.6)	−0.05	8.7 (0.3–24.6)	10.6 (0.9–24.9)	0.09
Albumin level, g/dl	3.2 (2.7–3.7)	3.1 (2.5–3.6)	−0.11	3.2 (2.5–3.7)	3.1 (2.5–3.6)	−0.09
Creatinine clearance^a^, ml/min	0.9 (0.7–1.3)	0.9 (0.7–1.4)	0.00	0.9 (0.7–1.3)	0.9 (0.7–1.5)	0.07
Lactate level, mmol/L	2.3 (1.4–3.8)	1.9 (1.2–3.1)	−0.23	2.3 (1.4–3.8)	2.2 (1.3–3.7)	−0.00
Base excess^a^, mEq/L	−1.1 (−4.0–1.2)	−1.1 (−3.8–1.1)	0.03	−0.6 (−3.9–1.2)	−1.3 (−4.5–1.0)	−0.07
PaO_2_/FiO_2_ ratio^a^	347 (267–401)	350 (282–414)	0.11	350 (267–408)	348 (271–414)	0.03
SOFA score	3 (1–5)	2 (1–4)	−0.09	3 (1–5)	2 (1–4)	−0.00
Hinchey classification			−0.48			0.00
I	1 (0.6)	179 (11.6)		1 (1.2)	14 (4.3)	
II	5 (6.1)	141 (9.2)		5 (6.1)	13 (4.0)	
III	31 (35.2)	619 (40.3)		31 (37.8)	113 (34.5)	
IV	51 (58.0)	596 (38.8)		45 (54.9)	188 (57.3)	
Operation time, min	195 (155–236)	152 (120–192)	−0.59	193 (155–221)	181 (141–226)	−0.05
Intraoperative transfusion	26 (29.5)	455 (29.6)	0.00	25 (30.5)	95 (28.9)	−0.03
Stoma	77 (87.5)	1318 (85.8)	−0.04	72 (87.8)	285 (86.9)	−0.02
Retractor	60 (68.0)	1160 (75.6)	0.16	57 (69.6)	220 (66.9)	−0.05

*Note*: Categorical variables are expressed as counts and percentages. Numeric variables are expressed as median (25th–75th percentiles).

Abbreviations: FiO_2_, Fraction of inspiratory oxygen; NPWT, negative‐pressure wound therapy; PaO_2_, Partial pressure of arterial oxygen; SMD, Standardized mean difference; SOFA, Sequential Organ Failure Assessment.

^a^ These variables were not included in the model for propensity score estimation.

### 3.3 Primary analysis

Before propensity score matching, the incidence rates of iSSI in the NPWT and control groups were 19 (21.6%) and 380 (24.8%), respectively (OR, 0.97; 95% CI, 0.88–1.06). After the propensity score matching, the incidence rates of iSSI in the NPWT and control groups were 18 (22.0%) and 115 (35.0%), respectively. The incidence of iSSI was significantly lower in the NPWT group than in the control group (OR, 0.52; 95% CI, 0.30–0.92). There was no significant difference in the secondary outcomes between the two groups. The length of hospital stay in the NPWT and control groups were 23 days (16, 43) and 29 days (19, 49) days, respectively, and the healthcare costs in the two groups were $18,500 (16,000, 43,000) and $19,900 (13,200, 30,900), respectively (Table 2).

**TABLE 2:** Results of the analysis for the study outcomes by propensity score matching

Outcomes	NPWT (*n* = 82)	Control (*n* = 328)	Odds ratio (95% CI)	Difference (95% CI)	*p*‐value
Primary outcome					
Incisional SSI	18 (22.0)	115 (35.0)	0.52 (0.30–0.92)		0.026
Secondary outcomes					
Hospital stay (days)	23 (16, 43)	29 (19, 49)		−3 (−12–6)	0.569
Healthcare cost ×1000 USD	18.5 (13.9, 29.0)	19.9 (13.2, 30.9)		0.7 (−4.3–5.7)	0.782
In‐hospital death	4 (4.9)	34 (10.4)	0.44 (0.15–1.29)		0.135
Complications except for SSI	28 (34.1)	127 (38.6)	0.82 (0.50–1.37)		0.460

*Note*: Continuous variables are expressed as median (25th–75th percentiles), whereas categorical variables are presented as number (%). The level of significance was defined as *p* < 0.05.

Abbreviations: CI, Confidence interval; NPWT, negative‐pressure wound therapy; SSI, Surgical site infection.

### 3.4 Sensitivity analysis

All patients were evaluated using the logistic regression analysis. As all the variance inflation factors for the variables were lower than 2, the issue of multicollinearity was eliminated in the model. The results indicated the tendency of NPWT to decrease the incidence of iSSI (19 [21.6%] in the NPWT group and 380 [4.8%] in the control group; OR, 0.92; 95% CI, 0.84–1.01; *p* = 0.079).

### 3.5 Subgroup analysis

**FIGURE 2:**
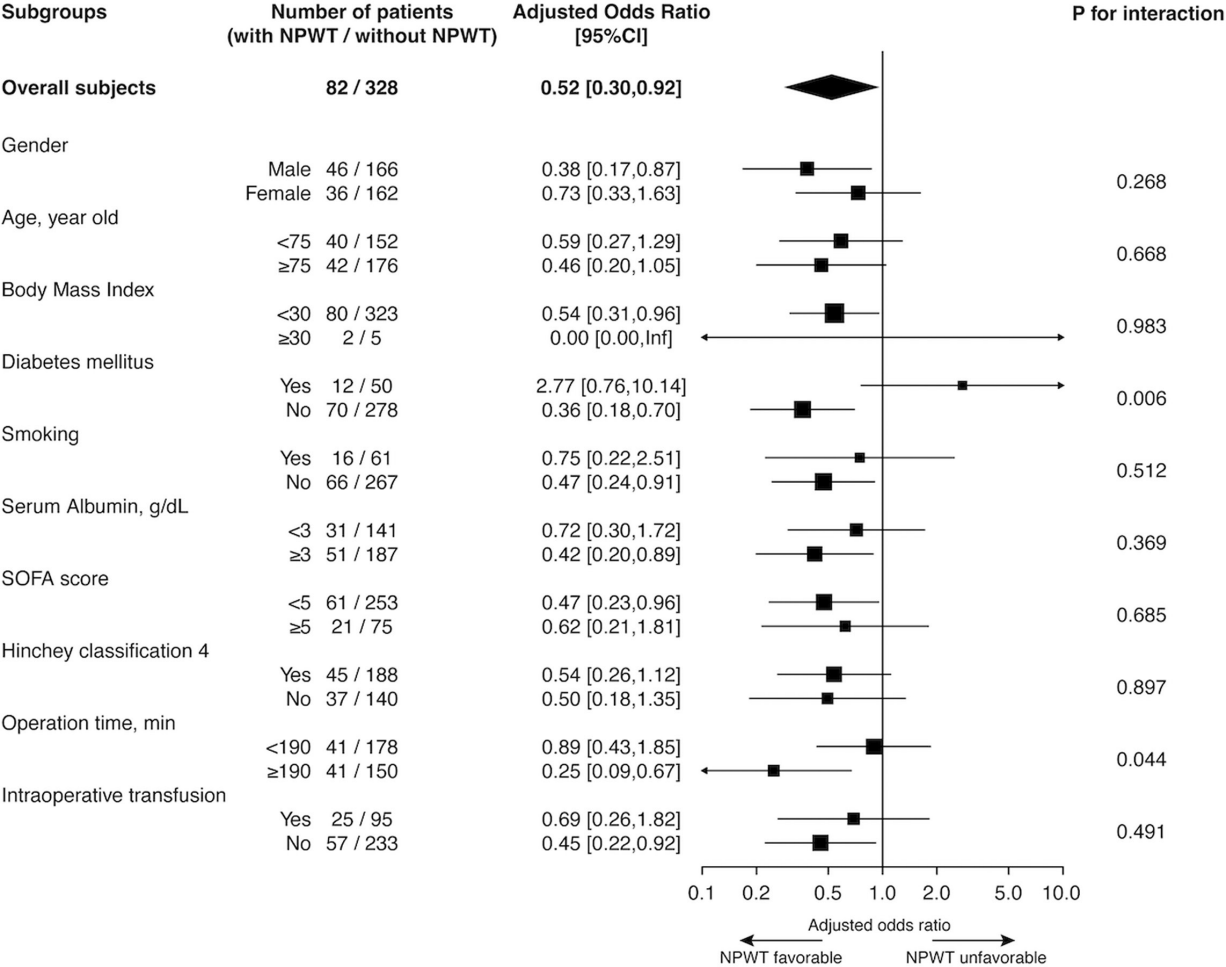
Results of subgroup analysis for the primary outcome. ORs for the incidence of incisional surgical site infection (95% CI) in each subgroup and *p*‐values for the interaction between subgroups are shown. OR: Odds ratio, CI: Confidence interval, SOFA: Sequential organ failure assessment

The results of the subgroup analysis are presented in Figure 2. The presence or absence of diabetes mellitus and operative time had a significant impact on the effect of NPWT. Patients without diabetes mellitus and those who underwent a longer operation showed the favorable effects of NPWT.

## 4 DISCUSSION

This nationwide multicenter study showed that the prophylactic use of NPWT and delayed sutures was significantly associated with a lower incidence of iSSI after emergency surgery for colorectal perforation. Several previous, smaller studies have suggested the prophylactic effect of NPWT for emergency contaminated surgery.^18^, ^19^, ^20^ However, to the best of our knowledge, the present study is the first large‐scale study to demonstrate the prophylactic effect of NPWT against iSSI after highly contaminated emergency laparotomies. Moreover, specific subpopulations that might likely benefit from NPWT were explored. This could contribute to the suggestion of adequate indications for the prophylactic use of NPWT.

A previous randomized controlled trial did not show the benefit of prophylactic use of NPWT for a decrease in SSI after elective colorectal surgery.^21^ Nevertheless, in the present study, we focused on patients who underwent emergency surgery for colorectal perforation, who were supposed to be one of the most affected candidates owing to the nature of the highly contaminated surgical field that causes iSSI. The difference in the target population could be a reason for the difference in the results between our study and the previous randomized controlled trial.

The use of NPWT could lead to longer hospitalizations than conventional treatments.^22^ In addition, NPWT requires additional healthcare costs; thus, guidelines recommend that the adaptation of NPWT should be carefully considered.^10^ On the other hand, several previous studies insisted that NPWT could decrease hospitalization and healthcare costs, reducing wound healing time.^23^, ^24^, ^25^ In the present study, the prophylactic use of NPWT was not associated with an increase in hospitalization and healthcare costs, possibly because of the lower incidence of iSSI, which led to longer hospital stay and higher healthcare costs. This result might lead to easier access for the prophylactic use of NPWT from the viewpoint of healthcare costs.

Diabetes mellitus, smoking, and undernutrition are patient‐level risk factors for iSSI, whereas contaminated‐surgery and long operative time are surgical‐level risk factors for iSSI.^26^ Generally, patients with these risks of iSSI are considered to be more appropriate candidates for the prophylactic use of NPWT.^10^, ^27^ The present study showed that patients who underwent a longer operation were likely to benefit from the favorable effects of NPWT. Interestingly, patients without diabetes mellitus exhibited a more favorable prophylactic effect of NPWT than those with diabetes mellitus. In addition, a significant benefit of prophylactic NPWT was not observed in current smokers and patients with lower serum albumin levels, as compared to that in non‐current smokers and those with normal serum albumin levels. Patient‐level risk factors for the appropriate indication for the prophylactic use of NPWT should be further studied in the future.

Several limitations of this study should be considered when interpreting its results. First, our intervention procedure involved a combination of prophylactic NPWT and delayed suturing. The independent effect of delayed suture compared to primary suture for iSSI has already been known,^28^ which could have led to an overestimation of the effect of prophylactic NPWT in the present study. Although a previous small study showed that there was no difference in wound complications between open NPWT and closed NPWT,^29^ additional studies with large populations are needed to evaluate the difference between the two approaches. Second, this study had a retrospective design, and the data were collected from each facility. Thus, the method of general wound management, such as dressing and washing, was not protocolized and was determined based on the facilities or preference of the attending surgeons. The meticulousness of wound management might be different among the cases that could influence the results. Furthermore, although the diagnosis of iSSI was made based on the CDC standard,^13^ it was subjectively performed by the attending surgeon. This might have resulted in some risk of bias. Third, as the prophylactic use of NPWT was not sufficiently common during the study period, many patients without NPWT dropped out, via the matching process, owing to the small number of patients who received NPWT. A study assessing a larger sample size of patients treated with NPWT is needed in the future. Finally, nationwide data from Japan were retrospectively analyzed in this study. Thus, residual confounding was unavoidable, and the generalizability of the results is limited owing to international differences in medical care and systems.

## 5 CONCLUSIONS

The prophylactic use of NPWT with delayed sutures was associated with a lower incidence of iSSI after emergency surgery for colorectal perforation, without an increase in hospital stay and healthcare costs. Patients without diabetes mellitus or with longer operative times (≥190 min) might benefit from NPWT. Further prospective analyses with a larger number of patients are required.

## DISCLOSURE

Author contributions: KN drafted and revised the manuscript, contributed to the study conception and design, performed statistical analysis and data interpretation, and accepted the responsibility for the conduct of research, final approval, and study supervision. AE revised the manuscript, contributed to study concept and design, and performed statistical analysis and data interpretation. AH, SS, RK, SK, WI, MN, and NT conducted data collection and interpretation and revised the manuscript. YO provided critical advice on the drafting of the manuscript. All authors have read and approved the final manuscript.

Funding information: The authors received the project study fund from the Japanese Society for Abdominal Emergency Medicine.

Conflict of interest: The authors declare no conflict of interests for this article.

Ethics statements: This study was conducted in accordance with the principles of the Declaration of Helsinki and was approved by the ethics committee of the Tokyo Medical and Dental University (approval no.: M2020‐125).

## Supporting information


Table S1

Table S2
