# Supplementary material for: Prophylactic effect of negative‐pressure wound therapy and delayed sutures against incisional‐surgical site infection after emergency laparotomy for colorectal perforation: A multicenter retrospective cohort study
Source: Ann Gastroenterol Surg. 2022 Nov 27;7(3):441–9. doi: 10.1002/ags3.12643 (PMC10154815; doi:10.1002/ags3.12643)
Supplement: Supplementary file 1 — Table S1 [file AGS3-7-441-s001.docx]

| **TABLE S1** The number of cases in each facility | | |
| --- | --- | --- |
| **Facility Number** | **NPWT case** | **Control case** |
| 1 | 0 | 19 |
| 2 | 0 | 162 |
| 3 | 0 | 14 |
| 4 | 0 | 30 |
| 5 | 0 | 62 |
| 6 | 0 | 36 |
| 7 | 0 | 48 |
| 8 | 0 | 14 |
| 9 | 0 | 40 |
| 10 | 0 | 34 |
| 11 | 2 | 19 |
| 12 | 0 | 6 |
| 13 | 0 | 9 |
| 14 | 0 | 23 |
| 15 | 0 | 10 |
| 16 | 0 | 21 |
| 17 | 0 | 6 |
| 18 | 31 | 4 |
| 19 | 0 | 50 |
| 20 | 8 | 42 |
| 21 | 6 | 33 |
| 22 | 0 | 21 |
| 23 | 0 | 5 |
| 24 | 0 | 36 |
| 25 | 2 | 69 |
| 26 | 0 | 26 |
| 27 | 0 | 5 |
| 28 | 0 | 12 |
| 29 | 0 | 30 |
| 30 | 0 | 11 |
| 31 | 1 | 28 |
| 32 | 1 | 108 |
| 33 | 0 | 4 |
| 34 | 0 | 36 |
| 35 | 0 | 7 |
| 36 | 0 | 41 |
| 37 | 0 | 89 |
| 38 | 0 | 54 |
| 39 | 0 | 22 |
| 40 | 0 | 28 |
| 41 | 0 | 18 |
| 42 | 0 | 39 |
| 43 | 0 | 26 |
| 44 | 0 | 19 |
| 45 | 0 | 34 |
| 46 | 34 | 10 |
| 47 | 2 | 46 |
| 48 | 1 | 29 |
| Note: NPWT, negative-pressure wound therapy. | | |
