# Supplementary material for: Prophylactic effect of negative‐pressure wound therapy and delayed sutures against incisional‐surgical site infection after emergency laparotomy for colorectal perforation: A multicenter retrospective cohort study
Source: Ann Gastroenterol Surg. 2022 Nov 27;7(3):441–9. doi: 10.1002/ags3.12643 (PMC10154815; doi:10.1002/ags3.12643)
Supplement: Supplementary file 2 — Table S2 [file AGS3-7-441-s002.docx]

| **TABLE S2** The all-patient non-imputed demographic data | | | |  |
| --- | --- | --- | --- | --- |
| **Variables** |  | **Variables** |  | |
| Age, years | 75 (65–83) | SOFA score | 2 (1–4) | |
| Male | 793 (48.9) | Hinchey classification |  | |
| Body Mass Index | 22 (19–24) | Ⅰ | 166 (11.2) | |
| Performance status ≥ 3 | 254 (15.9) | Ⅱ | 136 (9.1) | |
| Smoking | 302 (19.8) | Ⅲ | 595 (40.0) | |
| Diabetes mellitus | 215 (13.2) | Ⅳ | 590 (39.7) | |
| Immunosuppressive therapy | 162 (10.0) | The cause of perforation |  | |
| Cancer-bearing | 404 (24.8) | Diverticulitis | 589 (36.4) | |
| Perioperative vital signs |  | Tumor | 383 (23.7) | |
| Median blood pressure, mmHg | 90 (78–102) | Coprostasis | 211 (13.1) | |
| Heart rate, beats/min | 94 (80–110) | Iatrogenicity | 123 (7.6) | |
| Respiratory rate, beats/min | 21 (18–26) | Trauma | 24 (1.5) | |
| Glasgow coma scale | 15 (15–15) | Others | 286 (17.7) | |
| Perioperative blood exam results |  | Operation time, min | 154 (121–195) | |
| White blood cell count, 10^3^/µL | 8.1 (4.2–13.2) | Intraoperative transfusion | 482 (29.7) | |
| C-reactive protein, mg/dL | 9.4 (0.7–21.4) | Stoma | 1394 (85.9) | |
| Albumin level, g/dL | 3.1 (2.5–3.6) | Retractor | 1019 (73.8) | |
| Creatinine clearance, mL/min | 0.9 (0.7–1.3) |  |  | |
| Lactate level, mmol/L | 1.9 (1.2–3.2) |  |  | |
| Base excess, mEq/L | -1.0 (-3.8–1.1) |  |  | |
| PaO_2_/FiO_2_ ratio | 356 (289–415) |  |  | |
| Note: Categorical variables are expressed as counts and percentages. Numeric variables are expressed as median (25^th^–75^th^ percentiles).  PaO_2_, Partial pressure of arterial oxygen; FiO_2_, Fraction of inspiratory oxygen; SOFA, Sequential Organ Failure Assessment. | | | |  |
